# Hypothalamic Inflammation as a Critical Link Between Metabolic Dysfunction and Impaired Endometrial Receptivity in Obese Polycystic Ovary Syndrome

**DOI:** 10.33549/physiolres.935770

**Published:** 2026-06-01

**Authors:** Chang SUN, Yapeng HAN, Zimeng PAN, Jing LI, Xianzhuo CHENG, Miao SUN, Ying WANG, Hongying KUANG

**Affiliations:** 1 Heilongjiang University of Chinese Medicine, Harbin, China; 2 The Sixth Department of Acupuncture, The First Affiliated Hospital of Heilongjiang University of Chinese Medicine, Harbin, China; 3 Department of Obstetrics and Gynecology, Shunyi Hospital, Beijing Hospital of Chinese Medicine, Beijing, China; 4 Department of Gynecology, The Second Affiliated Hospital of Tianjin University of TCM, Tianjin, China; 5 Second Department of Gynecology, The First Affiliated Hospital of Heilongjiang University of Chinese Medicine, Harbin, China

**Keywords:** Polycystic ovary syndrome (PCOS), Hypothalamic inflammation, Insulin resistance, Endometrial receptivity, TLR4/IKKβ pathway

## Abstract

Obese polycystic ovary syndrome (PCOS) involves insulin resistance (IR) and impaired endometrial receptivity, potentially driven by hypothalamic inflammation. We investigated if inhibiting the hypothalamic Toll-like receptor 4 (TLR4)/IκB kinase β (IKKβ) pathway could ameliorate IR and improve endometrial receptivity in an obese PCOS rat model. An obese PCOS rat model was established using letrozole and high-fat diet. Rats were divided into control, PCOS, and three intervention groups: PCOS + intracerebroventricular (i.c.v.) TAK242 (central TLR4 inhibitor), PCOS + intraperitoneal (i.p.) TAK242 (systemic inhibitor), and PCOS + Metformin. We assessed hypothalamic inflammation (TLR4/IKKβ pathway), metabolic parameters (body weight, OGTT, ITT, HOMA-IR), systemic inflammation, and reproductive phenotypes. Endometrial receptivity was evaluated at pregnancy day 6 by uterine morphology, histology, pinopode development (SEM), and receptivity markers (BMP2, VEGF, IGFBP-1). The obese PCOS model successfully induced hypothalamic inflammation (activated TLR4/IKKβ pathway), systemic inflammation, profound IR, and impaired endometrial receptivity (poor decidualization, sparse pinopodes, suppressed receptivity markers). Central (i.c.v.) TAK242 administration was most effective, significantly suppressing the hypothalamic TLR4/IKKβ pathway, attenuating weight gain, normalizing insulin sensitivity, and, critically, restoring endometrial receptivity. Metformin also improved IR and receptivity, but was less potent at inhibiting the hypothalamic TLR4/IKKβ pathway. Systemic (i.p.) TAK242 showed the least efficacy, particularly on metabolic and endometrial outcomes. Hypothalamic inflammation (TLR4/IKKβ pathway) appears to be a critical mechanism linking IR and impaired endometrial receptivity in obese PCOS. Targeted central inhibition was more effective than systemic inhibition, suggesting this pathway is a novel therapeutic target.

## Introduction

Polycystic ovary syndrome (PCOS) is a prevalent endocrine disorder that adversely affects female reproductive health and the endocrine system. According to statistics, approximately 50 % of PCOS patients suffer from obesity, and 65–70 % of them exhibit insulin resistance (IR) symptoms [1]. These metabolic dysregulations are significant risk factors that contribute to the exacerbation of reproductive disorders. Patients with PCOS have a significantly increased risk of spontaneous abortion in the early stages of pregnancy [2], and the incidence of adverse pregnancy outcomes is closely associated with metabolic disorders [3]. Maintaining normal physiological blood sugar levels is crucial for ensuring the homeostasis of energy metabolism within the uterus during the embryo implantation stage. IR can impair uterine glucose utilization, thereby causing impaired growth and development of the endometrium, decreased receptivity of the endometrium, and an increased rate of miscarriage [4]. The characteristic feature of PCOS is a persistent low-level inflammatory state, in which the initial activation of the inflammatory response originates in the hypothalamus [5]. Abnormal activation of microglia within the hypothalamus can lead to hypothalamic inflammation [6], and this inflammation is now recognized as a key factor in triggering metabolic disorders [7]. Given that the hypothalamus plays a critical role in regulating reproductive functions, an increasing number of studies have proposed that hypothalamic inflammation may serve as the underlying pathophysiological basis for the metabolic, reproductive, and clinical heterogeneity observed in PCOS [5].

The Toll-like receptor 4 (TLR4)/IκB kinase β (IKKβ) signaling pathway plays a crucial role in the induction of hypothalamic inflammation. TLR4 is a transmembrane receptor that is mainly expressed on innate immune cells in the central nervous system (CNS), including endothelial cells, microglia, specific astrocytes, and mature neurons [8]. The ligand binds to TLR4 and its related molecules, thereby triggering the production of proinflammatory cytokines through intracellular signaling pathways dependent on or independent of myeloid differentiation factor 88 (MyD88), ultimately leading to an inflammatory response in the hypothalamus [9]. IKKβ is a key downstream component of the MyD88-dependent signaling pathway [10]. The research conducted by Purkayastha *et al*. [11] demonstrated that by injecting a plasmid encoding an inhibitory protein of IKKβ, namely IkBα, into the ventricles of the brain, it is possible to alleviate the glucose intolerance and insulin resistance phenomena observed in the hypothalamus caused by endoplasmic reticulum stress, by inhibiting the IKKβ/nuclear factor-κB (NF-κB) signaling pathway. Therefore, the hypothalamic inflammation caused by the TLR4/IKKβ pathway may lead to a decrease in the receptivity of the endometrium in patients with polycystic ovary syndrome by regulating the insulin and glucose metabolism throughout the body.

In this study, our aim was to investigate the role of hypothalamic inflammation by studying the effects of targeted inhibition of the TLR4/IKKβ pathway on IR and endometrial receptivity in obese PCOS rats. This research aimed to elucidate the potential mechanisms for alleviating the metabolic and reproductive disorders associated with PCOS.

## Materials and Methods

### Animals and ethical approval

A total of 140 female and 15 male specific pathogen-free (SPF)-grade Sprague-Dawley (SD) rats (10 weeks old, 180–200 g; and 11–12 weeks old, 250–300 g, respectively) were obtained from the Experimental Animal Center at Heilongjiang University of Chinese Medicine (Harbin, China). Animals were housed in a controlled environment (22–25 °C, 12:12 h light:dark cycle) with *ad libitum* access to food and water. All experimental protocols were approved by the Experimental Animal Ethics Committee of Heilongjiang University of Chinese Medicine (reference number: 2023102708) and adhered to the regulations of the International Association for the Study of Pain.

### Experimental design and animal groups

Following a 7-day acclimatization period, female rats were monitored daily *via* vaginal smear for two consecutive estrous cycles (approximately 10 days). Only rats exhibiting regular cycles were included and randomly assigned to one of eight groups: (1) Control, (2) intracerebroventricular (i.c.v.) Control, (3) intraperitoneal (i.p.) Control, (4) metformin (Met) Control, (5) PCOS, (6) i.c.v. (PCOS + i.c.v. TAK242), (7) i.p. (PCOS + i.p. TAK242), and (8) Met (PCOS + Metformin).

**Fig. 1:**
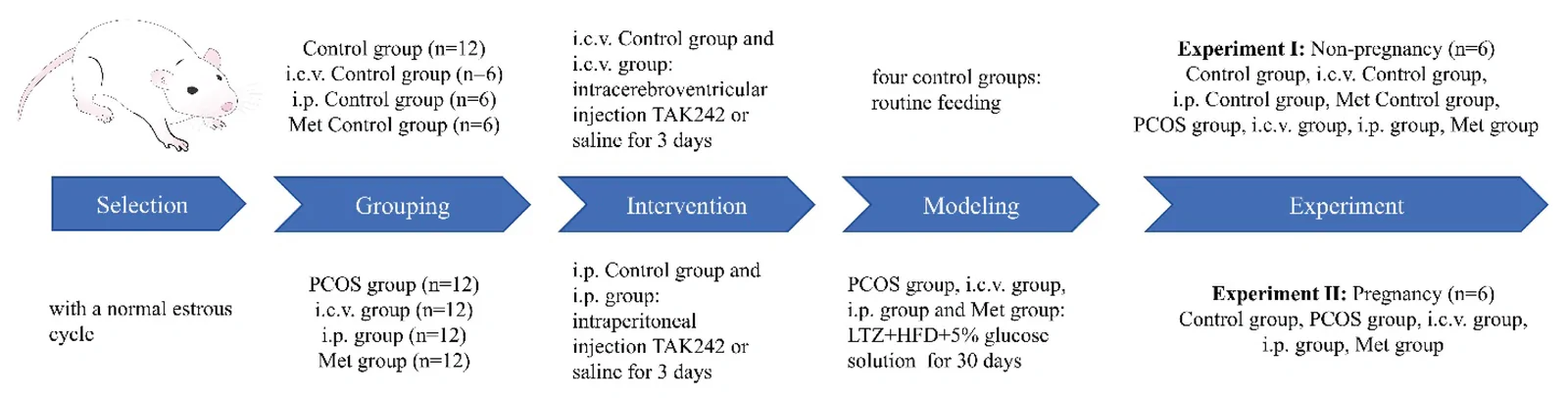
Experimental design to investigate the impact of hypothalamic inflammation inhibition on reproductive and metabolic functions in PCOS rats.

The study was conducted in two phases: a pre-pregnancy phase (to assess PCOS phenotype and metabolic parameters) and a pregnancy phase (to assess endometrial receptivity). Each of the eight primary groups consisted of 12 rats (n=12), which were further allocated to these phases (n=6 per group for the pre-pregnancy phase; n=6 per group for the pregnancy phase, with the exception of the control-vehicle groups which were not advanced to the pregnancy phase). The experimental timeline and group allocation are summarized in Fig. 1.

### Obese PCOS model induction

Rats in the PCOS, i.c.v., i.p., and Met groups were administered letrozole (1 mg/kg; Jiangsu Hengrui Pharmaceutical Co., Ltd, China) dissolved in 0.5 % sodium carboxymethyl cellulose solution (Zibo Hailan Chemical Co., Ltd, China) *via* oral gavage daily for 30 days. Concurrently, these rats were provided with a high-fat diet (HFD; XTHF45, Jiangsu Xietong Pharmaceutical Bio-engineering Co., Ltd, China) and a 5 % glucose drinking solution (Qingdao Haibo Biotechnology Co., Ltd, China). All control groups (Control, i.c.v. Control, i.p. Control, Met Control) received the maintenance diet (XTI01WC, Jiangsu Xietong) and purified water, along with daily gavage of the 0.5 % sodium carboxymethyl cellulose vehicle. The model was validated by daily vaginal smears, confirming persistent estrous (M/D phase) in PCOS rats versus regular cycles in controls.

#### Therapeutic interventions

##### Intracerebroventricular (i.c.v.) cannulation and administration

Prior to PCOS model induction, rats in the i.c.v. Control and i.c.v. groups were anesthetized *via* intraperitoneal injection of Zoletil® 50 (50 mg/kg; Virbac, French), a 1:1 mixture of tiletamine and zolazepam. Animals were fixed in a cerebral stereotaxic apparatus (ZH-B, Anhui Zhenghua Biological Instrument Equipment Co., Ltd, China). A guide catheter (M8020, Changsha Meyue BIO Co., Ltd, China) was implanted into the lateral ventricle using the following coordinates relative to bregma: 0.8 mm posterior, 1.5 mm lateral, and 3.5 mm ventral. The catheter was secured with dental cement (Shanghai Yuyan Scientific Instrument Co., Ltd, China).

Following a 7-day recovery period, infusions were performed continuously for 3 days using a 10 μl microsyringe (MC-10, Shanghai Gaoge Industry and Trade Co., Ltd, China). The i.c.v. group received the TLR4 inhibitor TAK242 (100 μM; HY-11109, MCE, USA) in a 10 μl volume, while the i.c.v. Control group received 10 μl of sterile normal saline [12]. Validation of i.c.v. cannulation was performed at the experiment’s conclusion by infusing 10 μl of 0.4 % Evans Blue dye (Bioss, Beijing, China) and confirming dye distribution within the ventricular system post-euthanasia. Data from rats with inaccurate injection sites were excluded. The catheter was then removed, the burr hole sealed with bone wax (Shenzhen Huayong Biotechnology Co., Ltd, China), and the incision sutured. Prophylactic penicillin was administered intramuscularly for 7 days post-surgery.

Intraperitoneal (i.p.) Administration: The i.p. Control and i.p. groups received intraperitoneal injections of either normal saline or TAK242 (3 mg/kg), respectively, for 3 consecutive days concurrent with the i.c.v. dosing schedule [13].

Metformin Administration: Following the 30-day modeling period, the Met Control and Met groups received daily oral gavage of normal saline or metformin (500 mg/kg), respectively, for 21 days [14].

### Pregnancy model and tissue collection

For the pregnancy phase, female rats from the Control, PCOS, i.c.v., i.p., and Met groups (n=6 per group) in the pre-luteal or luteal phase were mated with proven male breeders (2:1 female-to-male ratio) at 18:00. The presence of a vaginal plug or sperm in the vaginal smear at 07:00 the following morning confirmed pregnancy, designated as day 1 (P1). On P6, pregnant rats were euthanized *via* CO_2_ inhalation. Blood was collected *via* cardiac puncture, and hypothalamic, ovarian, and uterine tissues were immediately harvested, weighed, and processed for subsequent analyses.

### Histological and morphological analysis

Ovarian and uterine tissues were fixed in 4 % paraformaldehyde, processed for routine paraffin embedding, sectioned (5 μm), and subjected to hematoxylin and eosin (HE) staining (Baso Biotechnology, Zhuhai, China) for morphological evaluation.

For scanning electron microscopy (SEM), uterine tissues were fixed in 2.5 % glutaraldehyde, post-fixed with 1 % osmium tetroxide, dehydrated through an ethanol gradient, and critical-point dried (K850, Quorum, UK). Specimens were gold-coated using an ion sputtering instrument (MC1000, Hitachi, Japan) and imaged on an SEM (SU8100, Hitachi, Japan) to assess pinopode development.

### Metabolic function assessment

Serum levels of total cholesterol (TC) and triacylglycerol (TG) were quantified using a fully automated biochemical analyzer (AU480, Beckman Coulter, America).

An oral glucose tolerance test (OGTT) was performed on day 25 of modeling after a 12-hour fast. Rats received an oral glucose bolus (2.5 g/kg), and blood glucose was measured from the tail vein at 0, 30, 60, 90, and 120 min using a blood glucose analyzer (580, Yuwell Medical).

An insulin tolerance test (ITT) was conducted on day 28 after a 4-hour fast. Following baseline glucose measurement, rats were injected intraperitoneally with insulin (0.75 U/kg; United Laboratories, Zhuhai, China), and blood glucose was monitored at 0, 30, 60, 90, and 120 min.

Fasting insulin (FINS) was measured by ELISA, and the homeostatic model assessment of insulin resistance (HOMA-IR) was calculated as: FBG (mmol/l) × FINS (mU/l)/22.5.

### Enzyme-Linked Immunosorbent Assay (ELISA)

Serum levels of luteinizing hormone (LH, MM-0624R1), follicle-stimulating hormone (FSH, MM-70867R1), testosterone (T, MM-0577R1), estradiol (E2, MM-0575R1), interleukin-1β (IL-1β, MM-0047R1), tumor necrosis factor-α (TNF-α, MM-0180R1), and insulin (INS, MM-0587R1) were quantified using commercial kits (Meimian Industrial Co., Ltd, Jiangsu, China). Progesterone (P4, PP773; Beyotime) was also measured per manufacturer instructions. Uterine tissue homogenates were assayed for vascular endothelial growth factor (VEGF) and insulin-like growth factor binding protein 1 (IGFBP-1) (kits not specified).

### Western Blot (WB) analysis

Hypothalamic total protein was extracted using RIPA buffer and quantified *via* BCA assay (Beyotime, Shanghai, China). Samples (10 μg) were denatured, separated by SDS-PAGE (Solarbio, Beijing, China), and transferred to PVDF membranes (Millipore, America). Membranes were blocked for 2 h in 5 % non-fat milk in Tris-buffered saline with Tween-20 (TBST) and incubated overnight at 4 °C with primary antibodies (listed in Table S1). After incubation with HRP-conjugated secondary antibodies (Table S1) for 60 min, blots were visualized using ECL substrate (Beyotime) and imaged (Tanon 5200, Shanghai, China). Band intensities were quantified using Gelpro32 software and normalized to β-Actin.

### Immunohistochemistry (IHC)

Paraffin-embedded hypothalamic and uterine sections underwent deparaffinization, rehydration, and heat-induced antigen retrieval using citrate buffer (ZSGB-BIO, Beijing, China) or EDTA buffer (ZSGB-BIO). Endogenous peroxidase activity was quenched with 3 % H_2_O_2_, and non-specific binding was blocked with goat serum (Beyotime). Sections were incubated overnight at 4 °C with primary antibodies (Iba-1 or BMP2; Table S2), followed by HRP-conjugated secondary antibodies (FineTest or Sino Biological; Table S2) for 50 min. Signal was visualized using DAB solution (BSGB-BIO) and counterstained with hematoxylin.

### Immunofluorescence (IF)

Hypothalamic sections were prepared as for IHC, permeabilized with Triton X-100 (Beyotime), and blocked with 1 % BSA (Bioss, Beijing, China). Sections were co-incubated overnight at 4 °C with primary antibodies against Iba-1 and CD11b+CD1c (Table S3). Respective fluorescently conjugated secondary antibodies (FineTest and CST; Table S3) were applied for 50 min in the dark. Nuclei were counterstained with DAPI (Bioss) and sections mounted with an anti-fluorescence quencher (Solarbio).

### Statistical analysis

Data were analyzed using Graphpad 8.0 software and are presented as mean ± SEM. All analyses were performed with n=6 per group for each experimental phase. Normality was assessed using the Shapiro-Wilk test. For two-group comparisons, an unpaired Student’s *t*-test or Mann-Whitney U test was used. For multiple-group comparisons, one-way ANOVA followed by Tukey’s *post hoc* test, or the Kruskal-Wallis test followed by Dunn’s multiple comparisons test, was applied. A P-value <0.05 was considered statistically significant.

## Results

### Letrozole with high-fat diet induces an obese PCOS phenotype with metabolic dysfunction

**Fig. 2:**
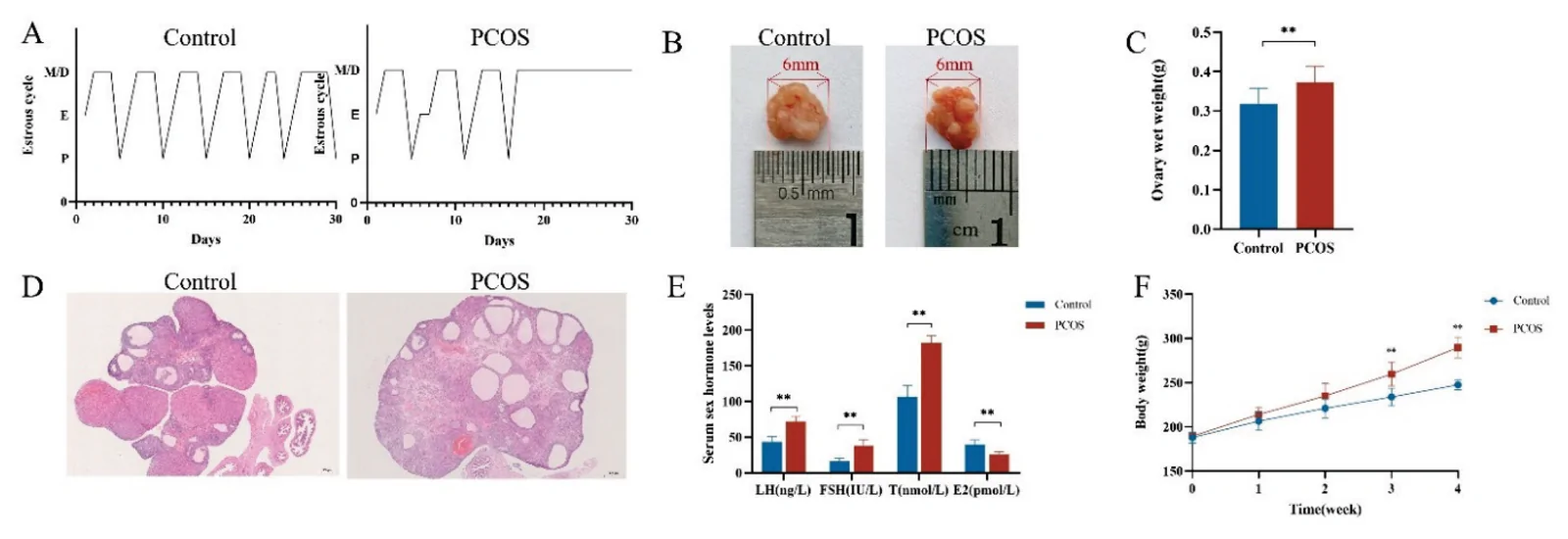
Comparison of related phenotypes between Control group and PCOS group. (**A**) Representative estrous cycles. (**B**) Gross morphology of ovaries. (**C**) Wet weight of ovary. (**D**) HE staining of ovarian tissue (×2.0 magnification). (**E**) Serum sex hormone levels. (**F**) Weekly body weight curve. P: proestrus; E: estrus; M/D: metestrus and diestrus; LH: luteinizing hormone; FSH: follicle-stimulating hormone; T: testosterone; E2: estradiol. Data are represented as the mean ± SEM (n=6). ** P<0.01 vs. Control group.

To validate our model, we first compared rats receiving the control diet (Control group) with those receiving letrozole and a high-fat diet (PCOS group). After 30 days, the PCOS group exhibited a persistent metestrus/diestrus phase, indicative of anovulation, whereas control rats maintained regular 4–5 day estrous cycles (Fig. 2A). Morphologically, ovaries from the PCOS group were enlarged, with a significantly greater wet weight (P<0.01) and an irregular, cystic surface compared to controls (Fig. 2B, C). Histological examination confirmed the PCOS phenotype, revealing numerous cystic and atretic follicles, a thin granulosa cell layer, and a reduced number of corpora lutea in the PCOS group, in stark contrast to the normal follicular development observed in controls (Fig. 2D).

This reproductive phenotype was accompanied by endocrine and metabolic disruptions. The PCOS group demonstrated significantly elevated serum LH, FSH, and Testosterone, and suppressed E2 levels (P<0.01 vs. Control; Fig. 2E). Furthermore, PCOS rats exhibited significantly greater body weight gain by week 3 (P<0.01; Fig. 2F). These data confirm the successful establishment of an obese, hyperandrogenic, anovulatory PCOS rat model.

### Central TLR4 inhibition markedly attenuates hypothalamic inflammation

**Fig. 3:**
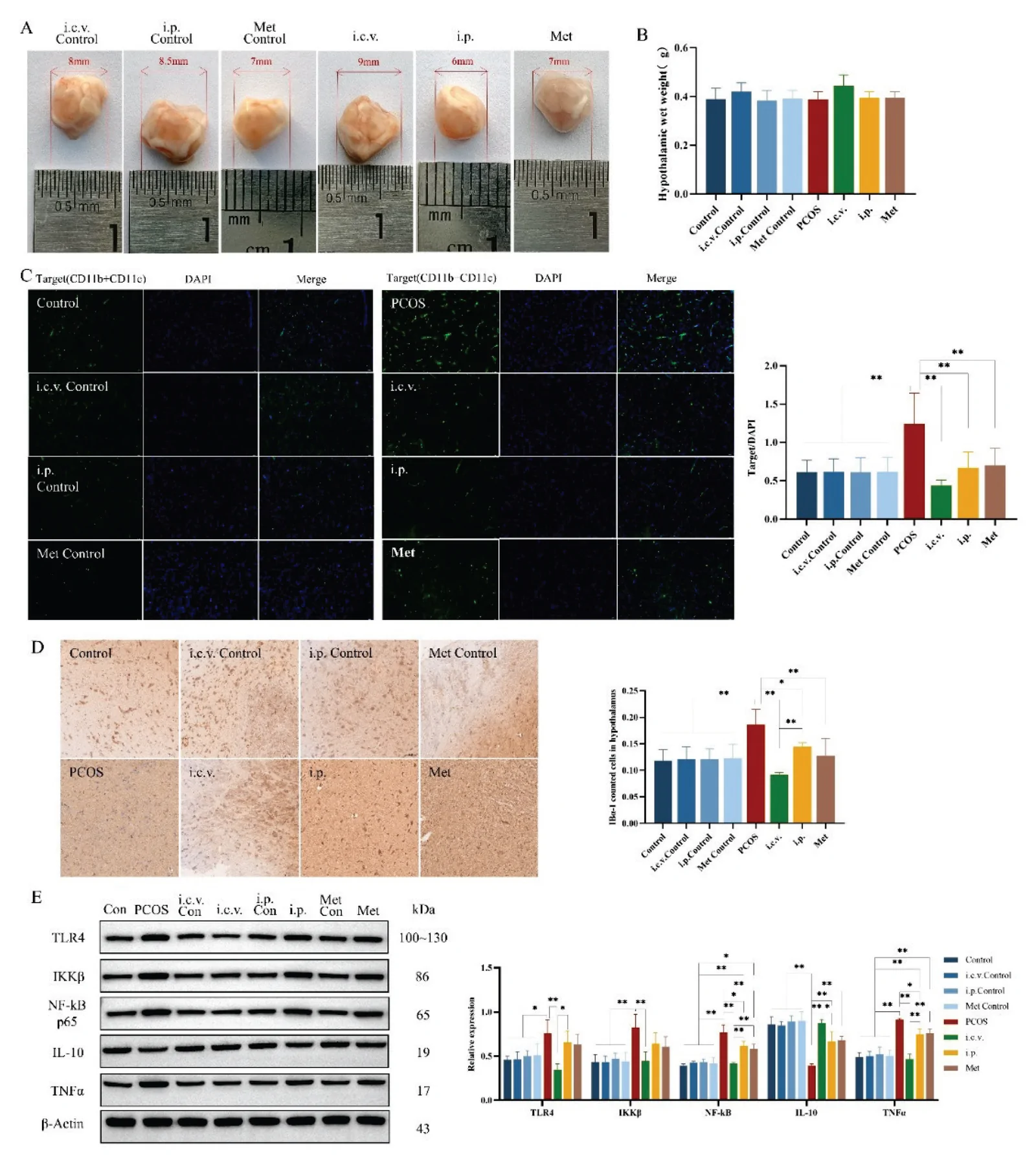
Effect of interventions on hypothalamic inflammatory response. (**A**) Gross morphology of hypothalamus. (**B**) Wet weight of hypothalamus. (**C**) Representative immunofluorescence of microglial activation (Iba-1/CD11c+CD1c co-localization; ×20.0 magnification). (**D**) Representative immunohistochemistry of Iba-1 expression (×20.0 magnification). (**E**) Relative protein expression of hypothalamic TLR4, IKKβ, NF-κB, IL-10, and TNF-α by Western blot. IBa-1: Ionized calcium-binding adapter molecule 1; TLR4: toll-like receptor 4; IKKβ: IκB kinase β; NF-κB: nuclear factor-κB; IL-10: interleukin 10; TNF-α: tumor necrosis factor α. Data are represented as the mean ± SEM (n=6). * P<0.05, ** P<0.01.

We next assessed inflammation within the hypothalamus. Gross morphology and wet weight of the hypothalamus did not differ among any experimental groups (Fig. 3A, B). However, immunofluorescence analysis revealed a striking increase in microglial activation (co-localization of Iba-1 and CD11b+CD1c) in the hypothalamus of the PCOS group compared to all four vehicle-control groups (P<0.01; Fig. 3C). IHC analysis further confirmed a significant increase in the expression of the microglial marker Iba-1 in PCOS rats (P<0.01; Fig. 3D).

At the molecular level, Western blot analysis demonstrated that the PCOS model triggered the hypothalamic TLR4/IKKβ pathway, evidenced by significantly upregulated protein expression of TLR4, IKKβ, NF-κB, and the pro-inflammatory cytokine TNF-α (P<0.01 vs. controls; Fig. 3E). Conversely, the anti-inflammatory cytokine IL-10 was significantly suppressed (P<0.01).

All three interventions mitigated this neuroinflammation. However, central (i.c.v.) administra-tion of the TLR4 inhibitor TAK242 was most effective, normalizing the expression of TLR4, IKKβ, NF-κB, and TNF-α to levels statistically indistinguishable from controls and significantly lower than those in the i.p. and Met groups (P<0.01; Fig. 3E). The i.c.v. TAK242 injection also restored IL-10 expression. While systemic (i.p.) TAK242 and metformin also reduced inflammatory markers compared to the PCOS group, their effect was less pronounced than central inhibition.

### Central and systemic TLR4 inhibition reduces systemic inflammation

**Fig. 4:**
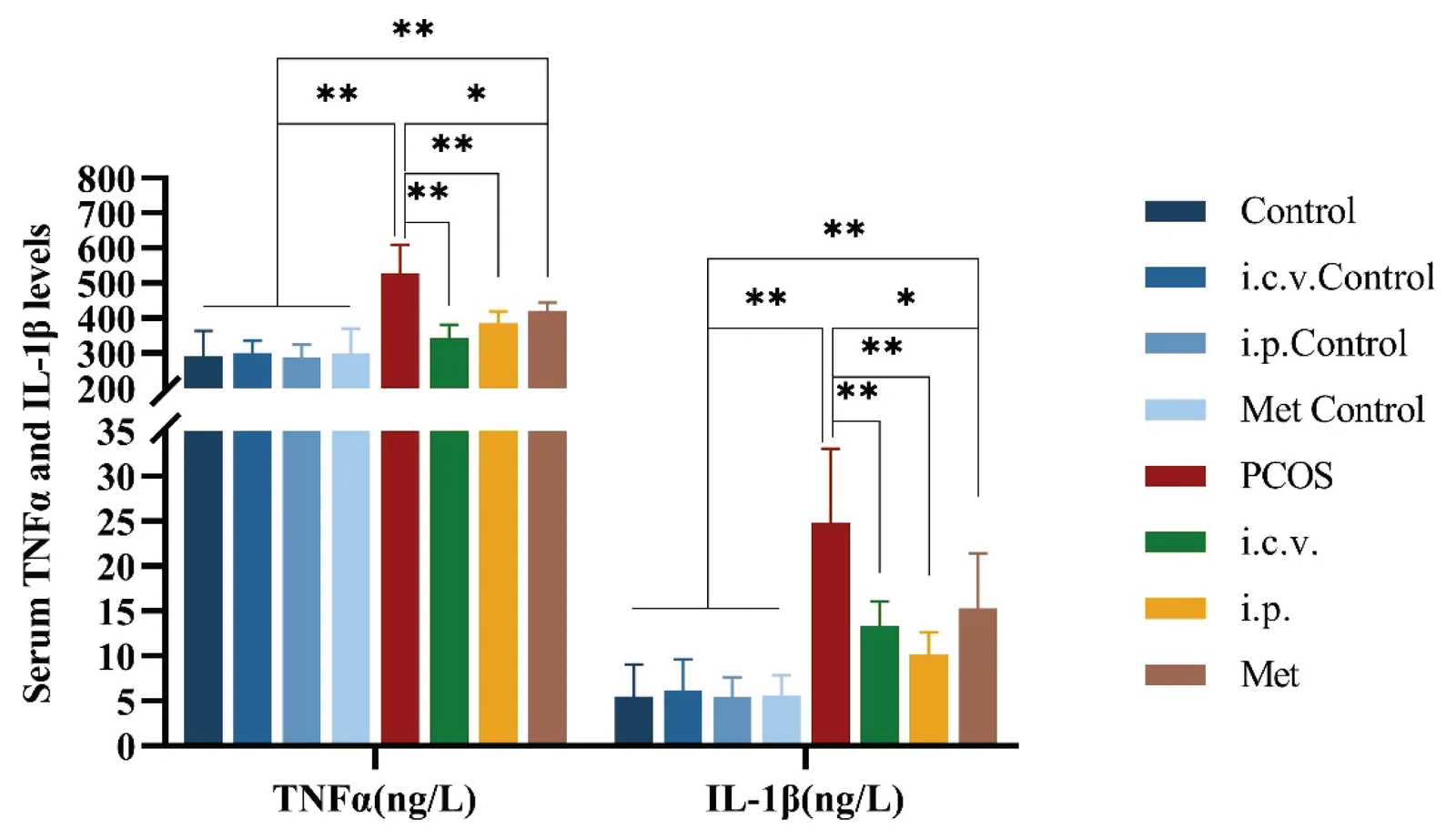
Effect of interventions on peripheral inflammatory response. Serum levels of TNF-α and IL-1β. TNF-α: tumor necrosis factor α; IL-1β: interleukin 1β. Data are represented as the mean ± SEM (n=6). ** P<0.01 vs. Control group; ^#^ P<0.05, ^##^ P<0.01 vs. PCOS group.

We then questioned if hypothalamic inflammation translated to a peripheral inflammatory state. Serum levels of the pro-inflammatory cytokines TNF-α and IL-1β were significantly elevated in the PCOS group compared to the four control groups (P<0.01; Fig. 4). Both i.c.v. and i.p. administration of TAK242, as well as metformin treatment, significantly reduced these circulating inflammatory markers compared to the untreated PCOS group (P<0.01 or P<0.05), indicating that both central and systemic interventions could ameliorate the systemic low-grade inflammation characteristic of PCOS.

### Targeted TLR4 inhibition and metformin ameliorate core PCOS phenotypes

**Fig. 5:**
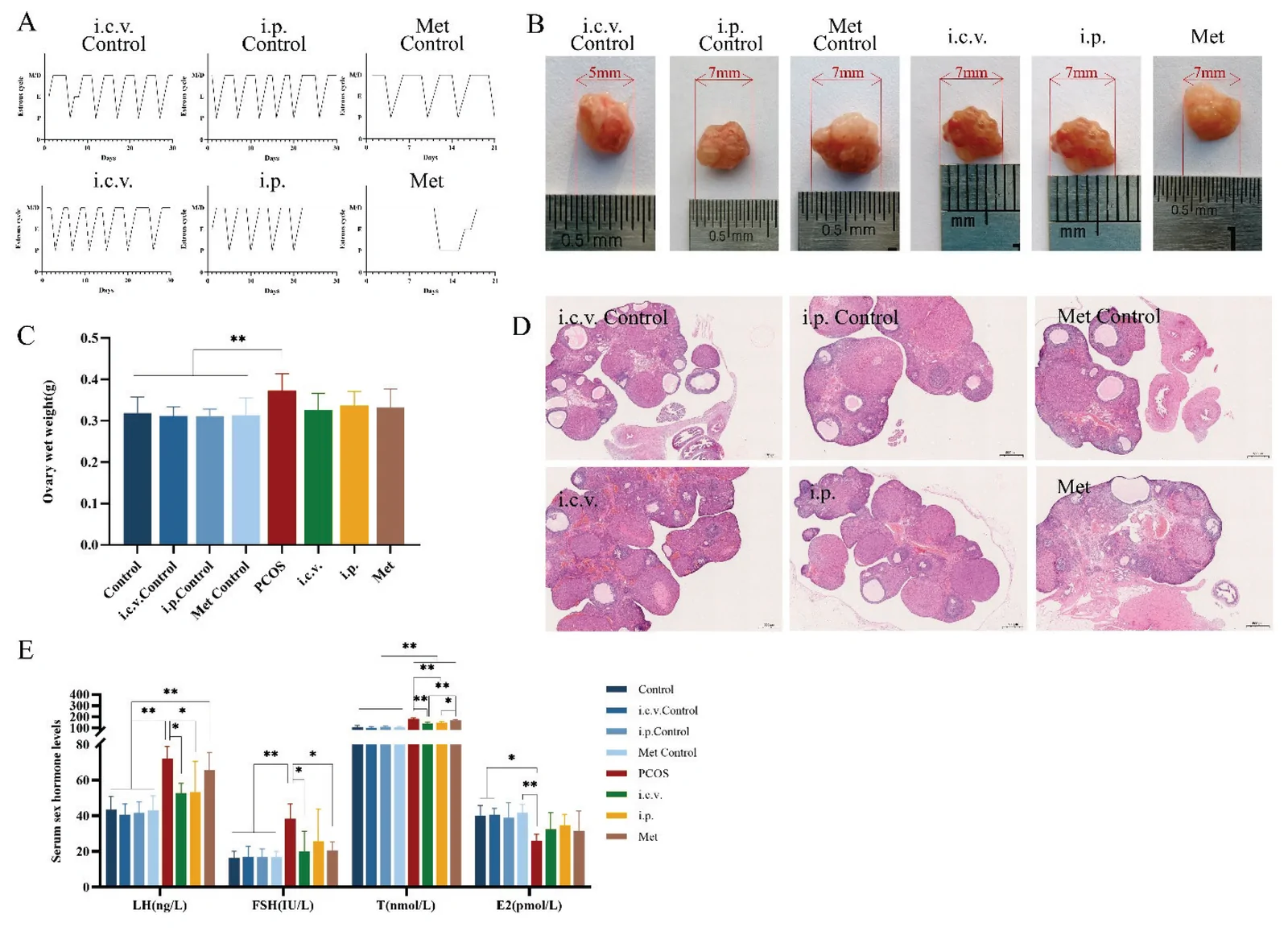
Effect of interventions on PCOS-related phenotypes. (**A**) Representative estrous cycles. (**B**) Gross morphology of ovaries. (**C**) Wet weight of ovary. (**D**) HE staining of ovarian tissue (×2.0 magnification). (**E**) Serum sex hormone levels. P: proestrus; E: estrus; M/D: metestrus and diestrus; LH: luteinizing hormone; FSH: follicle-stimulating hormone; T: testosterone; E2: estradiol. Data are represented as the mean ± SEM (n=6). * P<0.05, ** P<0.01.

Investigating the primary PCOS phenotype, we found that all interventions offered partial rescue (Fig. 5). The i.c.v. group showed a restored, albeit lengthened, estrous cycle, while the Met group also showed a trend towards cycle recovery. The i.p. group maintained normal cycles initially but progressed to the M/D stage later in the protocol (Fig. 5A).

All interventions improved ovarian morphology (Fig. 5B, D). Ovaries from the i.c.v., i.p., and Met groups were smoother, exhibited significantly reduced wet weight compared to the PCOS group (P<0.01; Fig. 5C), and displayed healthier follicular development with more corpora lutea and fewer cystic follicles upon HE staining (Fig. 5D). This morphological improvement was mirrored in the endocrine profile (Fig. 5E). Both i.c.v. and i.p. TAK242 administration significantly reduced the elevated serum LH and T levels (P<0.05 or P<0.01 vs. PCOS). Metformin also significantly reduced FSH levels (P<0.05 vs. PCOS).

### Central TLR4 Inhibition and Metformin Reduce Body Weight Gain and Improve Dyslipidemia

**Fig. 6:**
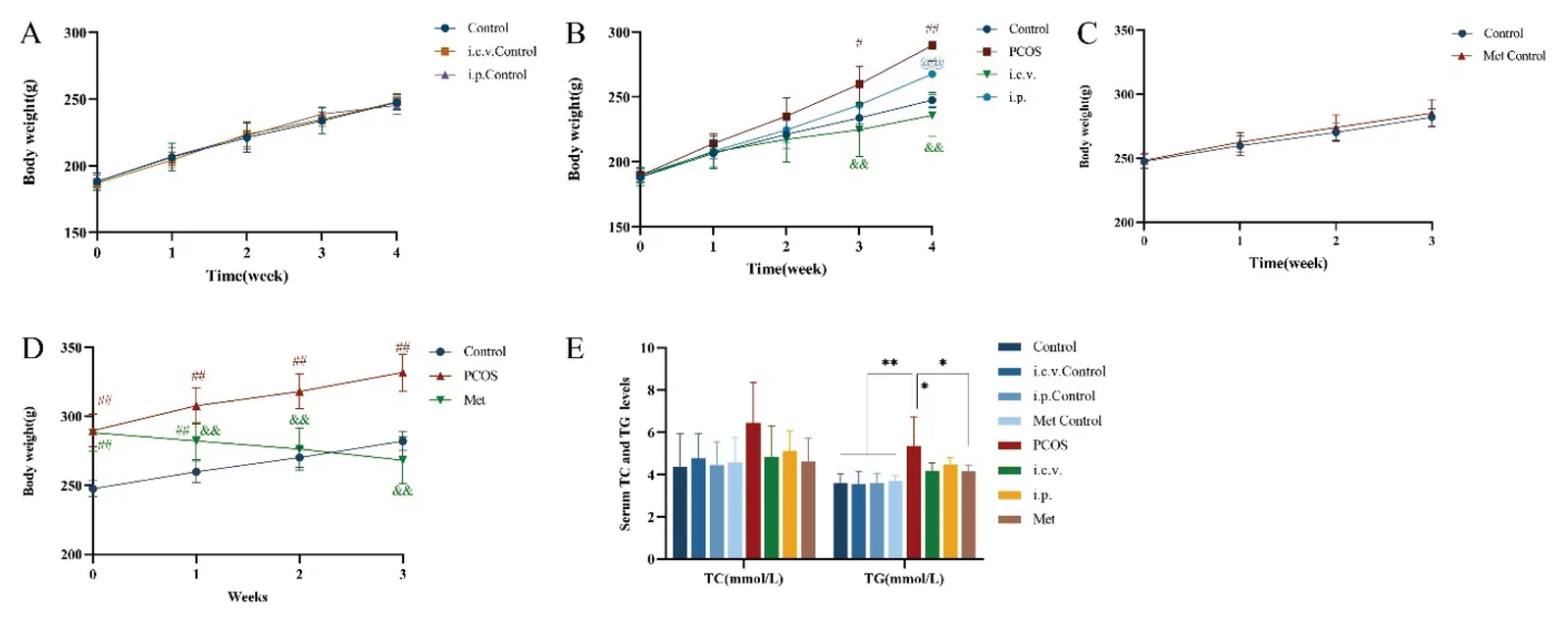
Effect of interventions on body weight and lipid metabolism. (**A–C**) Weekly body weight comparisons for control sub-groups. (**D**) Comparison of weekly body weight among the main experimental groups. (**E**) Serum lipid levels. TC: total cholesterol; TG: triglyceride. Data are represented as the mean ± SEM (n=6). ^##^ P<0.01 vs. Control group; ^&&^ P<0.01 vs. PCOS group; ^@@^ P<0.01 vs. i.c.v. group.

We next assessed the impact of these interventions on the obese phenotype. While all groups started at a similar weight, the PCOS group gained significantly more weight than controls (Fig. 6A–D). Central (i.c.v.) TAK242 administration significantly attenuated this weight gain by week 3, resulting in a body weight significantly lower than the PCOS and i.p. groups by week 4 (P<0.01; Fig. 6B). Metformin treatment also robustly controlled weight gain, with body weight remaining significantly lower than the PCOS group throughout the 3-week treatment period (P<0.01; Fig. 6D).

This metabolic improvement extended to dyslipidemia (Fig. 6E). The PCOS group exhibited significantly elevated serum TG levels compared to all control groups (P<0.01), a condition that was successfully reversed by both i.c.v. TAK242 and metformin (P<0.01 vs. PCOS). Serum TC levels were not significantly different among any groups.

### Central TLR4 inhibition and metformin restore insulin sensitivity and glucose homeostasis

**Fig. 7:**
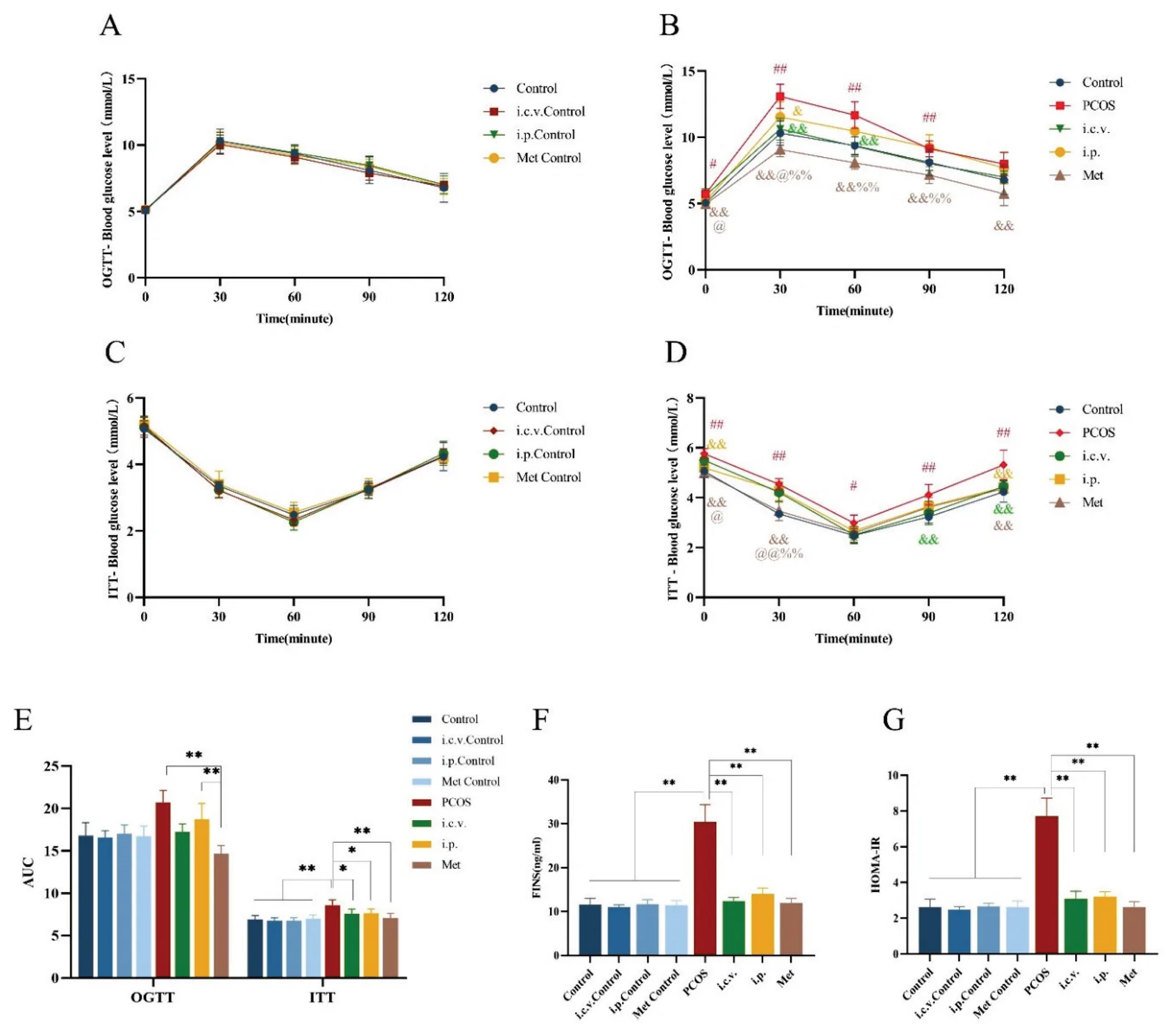
Effect of interventions on glucose and insulin metabolism. (**A**) OGTT for control sub-groups. (**B**) OGTT for main experimental groups. (**C**) ITT for control sub-groups. (**D**) ITT for main experimental groups. (**E**) OGTT and ITT area under the curve (AUC). (**F**) Fasting insulin (FINS). (**G**) HOMA-IR. OGTT: oral glucose tolerance test; ITT: insulin tolerance test; HOMA-IR: homeostatic model assessment of insulin resistance. Data are represented as the mean ± SEM (n=6). ^#^ P<0.05, ^##^ P<0.01 vs. Control group; ^&^ P<0.05, ^&&^ P<0.01 vs. PCOS group; ^@^ P<0.05, ^@@^ P<0.01 vs. i.c.v. group; ^%%^ P<0.01 vs. i.p. group.

The obese PCOS model demonstrated profound insulin resistance and impaired glucose tolerance (Fig. 7). All four control groups showed no differences in any metabolic tolerance tests (Fig. 7A, C, E). During the OGTT, PCOS rats displayed significantly elevated fasting blood glucose and exaggerated glycemic excursions, resulting in a significantly larger area under the curve (AUC) compared to controls (P<0.01; Fig. 7B, E). Metformin, serving as a positive control, was most effective in restoring glucose homeostasis, significantly lowering the glycemic curve at all time points and the overall AUC compared to the PCOS group (P<0.01). Central (i.c.v.) TAK242 administration also markedly improved glucose tolerance, significantly reducing blood glucose at 30 and 60 min (P<0.01 vs. PCOS). The i.p. TAK242 intervention had a modest, though significant, effect at the 60-minute time point (P<0.05).

The ITT confirmed severe insulin resistance in the PCOS group, which failed to lower blood glucose effectively in response to an insulin challenge, resulting in a significantly elevated AUC (P<0.01 vs. Control; Fig. 7D, E). All three interventions significantly improved insulin sensitivity, as shown by reduced glycemic levels and a significantly lower AUC compared to the PCOS group. This restoration of insulin sensitivity was corroborated by baseline measurements of fasting insulin (FINS) and HOMA-IR, which were significantly elevated in PCOS rats (P<0.01) and effectively reduced by i.c.v. TAK242, i.p. TAK242, and metformin treatments (P<0.01 vs. PCOS; Fig. 7F, G).

### Inhibition of hypothalamic inflammation enhances endometrial receptivity in pregnant PCOS rats

**Fig. 8:**
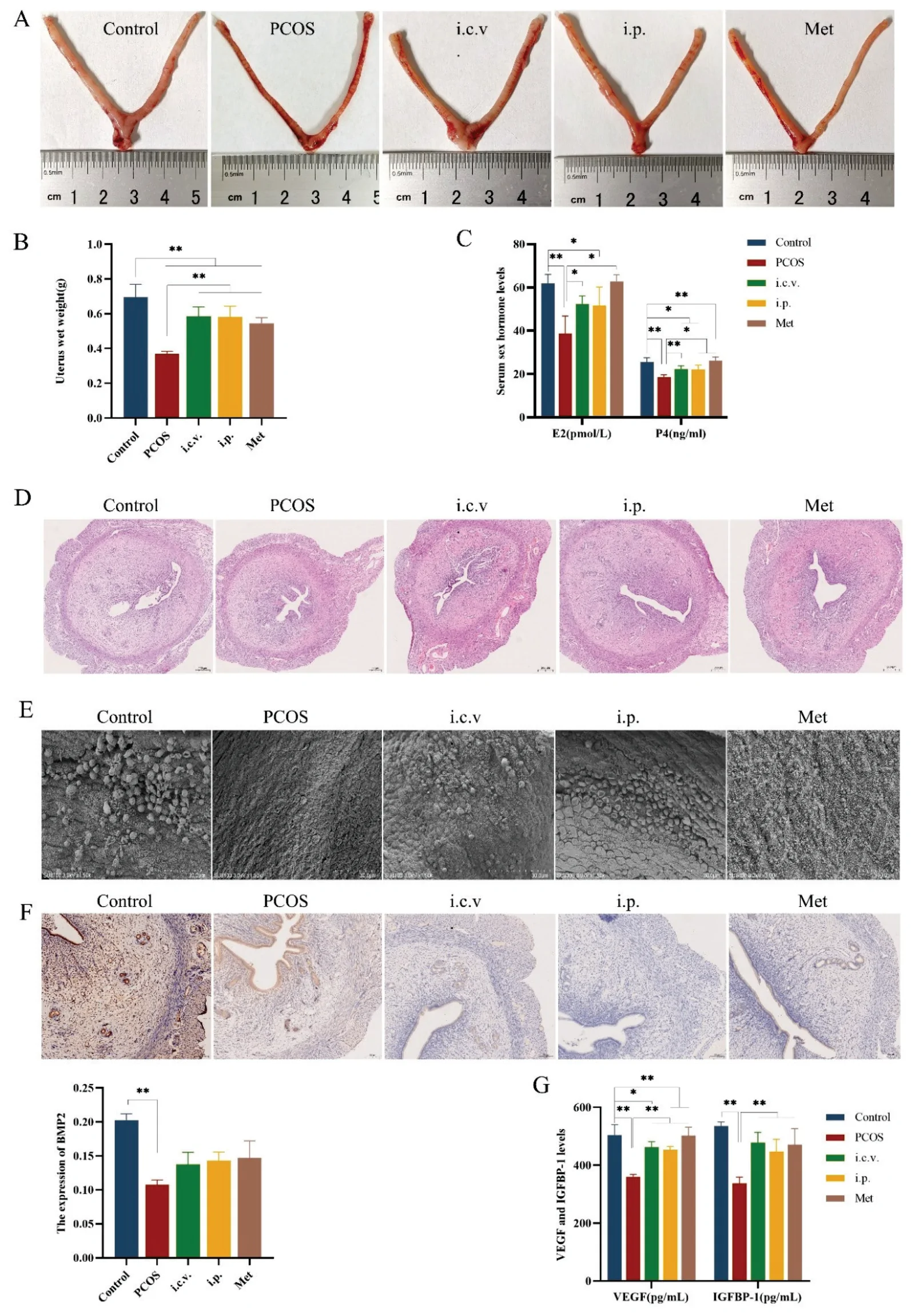
Effect of interventions on endometrial receptivity. (**A**) Gross morphology of P6 uteri. (**B**) Wet weight of uterus. (**C**) Serum E2 and P4 levels on P6. (**D**) HE staining of uterine implantation sites (×2.0 magnification). (**E**) SEM of endometrial pinopodes (×1500 magnification). (**F**) IHC of BMP-2 expression (×20.0 magnification). (**G**) Uterine tissue homogenate VEGF and IGFBP-1 levels. E2: estradiol; P4: progesterone; BMP2: Bone morphogenetic protein 2; VEGF: vascular endothelial growth factor; IGFBP-1: insulin-like growth factor binding protein 1. Data are represented as the mean ± SEM (n=6). * P<0.05, ** P<0.01 vs. Control group; ^#^ P<0.05, ^##^ P<0.01 vs. PCOS group.

Based on the pre-pregnancy results, we advanced the experimental groups (Control, PCOS, i.c.v., i.p., Met) to a pregnancy model to assess endometrial receptivity. On day P6, uteri from the Control group were visibly thickened with clear implantation sites, reflected in a high uterine wet weight (Fig. 8A, B). In contrast, uteri from the PCOS group were slender, lacked visible implantation humps, and had a significantly lower wet weight (P<0.01). All three interventions partially rescued this phenotype, resulting in uterine wet weights significantly greater than the PCOS group (P<0.01).

Hormonally, Control rats had high P4 levels, indicative of successful pregnancy support (Fig. 8C). PCOS rats showed significantly lower P4 levels. Central (i.c.v.) TAK242 and metformin treatments were most effective at restoring P4 levels, which were significantly higher than in the PCOS group (P<0.01 and P<0.05, respectively).

Histological analysis of implantation sites (Fig. 8D) revealed extensive decidualization, numerous glands, and robust vascularization in the Control group. The PCOS endometrium, however, showed minimal decidualization, few irregular glands, disordered stroma, poor vascularization, and significant inflammatory infiltration. All interventions improved this histology, with the i.c.v. TAK242 and Metformin groups showing the most pronounced decidualization and reduced inflammation.

SEM analysis of the endometrial surface (Fig. 8E) showed dense, mature pinopodes (villus protrusions) in the Control group, which are critical for implantation. These structures were sparse and underdeveloped in the PCOS group. Central (i.c.v.) TAK242 administration resulted in thick, well-developed pinopodes, whereas the i.p. and Met groups showed some improvement, but the pinopodes appeared atrophied.

Finally, we assessed molecular markers of receptivity (Fig. 8F, G). The expression of BMP2, VEGF, and IGFBP-1 – all crucial for implantation and decidualization – was significantly suppressed in the uteri of PCOS rats compared to controls (P<0.01). All three interventions (i.c.v., i.p., and Metformin) successfully and significantly upregulated the expression of these markers compared to the untreated PCOS group (P<0.01), consistent with the observed improvements in uterine morphology and pregnancy establishment.

## Discussion

In this study, we first established that a combination of letrozole and HFD successfully recapitulates the complex clinical phenotype of obese PCOS. Our model exhibited the requisite reproductive disruptions, including anovulatory cycles, polycystic ovarian morphology, and a hyperandrogenic endocrine profile. Critically, these reproductive deficits were accompanied by hallmark metabolic dysfunctions: significant weight gain, dyslipidemia, insulin resistance, and a state of low-grade systemic inflammation, aligning with the heterogeneity of the human condition.

A key finding of our study was the significant activation of the hypothalamic TLR4/IKKβ pathway, confirming the hypothalamus as a primary site of inflammation in this obese PCOS model. The expression of the microglial activation-specific protein Iba-1, as well as the relative expression levels of TLR4, IKKβ, NF-κB, and TNF-α, were all significantly higher than in controls. This aligns with extensive evidence linking HFDs to hypothalamic inflammation [15,16], which can be triggered within 72 h of HFD consumption, well before significant weight gain occurs [17]. This rapid activation may be mediated by mechanisms such as HFD-stimulated production of advanced glycation end products (AGEs) like CML, which in turn activate microglia *via* AGE receptors [6]. Our findings confirm that this central inflammatory pathway is active in our PCOS model and is not merely a non-specific histological artifact, as there were no differences in the macroscopic morphology or wet weight of the hypothalamus among groups [18].

To parse the specific role of this central inflammation, we compared targeted (i.c.v.) versus systemic (i.p.) inhibition of TLR4. TLRs are recognized mediators of metabolic inflammation [19], and the TLR4 signaling pathway is a key component of this process in the CNS [12]. Previous work has shown that HFDs trigger hypothalamic inflammation and metabolic dysfunction by activating this pathway, and that central inhibition can alleviate these changes [20,21]. Our results strongly support this. Intracerebroventricular injection of the TLR4 inhibitor TAK242 was demonstrably superior at suppressing the hypothalamic TLR4/IKKβ pathway and, in parallel, was more effective at reducing body weight, lowering serum TGs, and improving glucose tolerance compared to systemic (i.p.) administration. These results suggest that direct inhibition of hypothalamic inflammation provides a distinct metabolic advantage over peripheral inhibition alone. Interestingly, both central and systemic TAK242 administration similarly reduced *circulating* inflammatory cytokines (TNF-α, IL-1β) and were equally effective at partially rescuing the ovarian phenotype (follicular morphology, LH, and T levels). This suggests that while systemic inflammation contributes to the reproductive phenotype, the metabolic dysfunction is more tightly coupled to the inflammatory state within the hypothalamus.

We further contextualized these findings using metformin, a first-line therapy for PCOS that is known to improve insulin sensitivity and reduce inflammation [22]. Our results confirmed metformin’s efficacy; it effectively inhibited peripheral and central inflammation, improved the PCOS reproductive phenotype, and robustly controlled body weight and blood glucose. However, its inhibitory effect on hypothalamic inflammation was not as potent as direct i.c.v. TAK242 administration. Furthermore, our Western blot analysis suggests metformin may inhibit hypothalamic inflammation through a pathway independent of TLR4/IKKβ, as it did not significantly suppress their expression. Notably, while metformin was superior in lowering blood glucose during the OGTT, there was no significant difference in the primary insulin sensitivity metrics (FINS, HOMA-IR) between the metformin and i.c.v. TAK242 groups. Critically, when assessing the primary reproductive outcome of endometrial receptivity, central TLR4 inhibition *via* i.c.v. TAK242 was the most effective intervention, followed by metformin. Both treatments significantly improved uterine weight, hormonal support (P4 levels), decidualization, pinopode development, and the expression of receptivity markers (BMP2, VEGF, IGFBP-1) compared to the untreated PCOS group.

Adequate energy supply is a critical prerequisite for reproduction, and a complex feedback mechanism exists between metabolic signals and the HPG axis [23]. Both clinical and animal studies confirm that diseases associated with IR, such as PCOS, can lead to HPG axis dysfunction and impaired follicular development [24]. Indeed, IR and hyperinsulinemia are considered by some to be key factors in the pathogenesis of PCOS, driving androgen-dependent anovulation [25]. Our study supports this close relationship, demonstrating that blocking inflammatory signals specifically within the hypothalamus significantly improves IR [26]. By showing that this central intervention also yields the most significant improvement in endometrial receptivity, we provide evidence that hypothalamic inflammation may be a critical upstream driver of both the metabolic and reproductive pathologies in obese PCOS.

In conclusion, our findings demonstrate that hypothalamic inflammation, mediated at least in part by the TLR4/IKKβ pathway, is a critical driver of both metabolic and reproductive dysfunction in an obese PCOS rat model. This central inflammation contributes significantly to systemic insulin resistance and, in parallel, impairs endometrial receptivity, which may underlie the increased risk of poor pregnancy outcomes in PCOS. Our data suggest that targeted anti-inflammatory therapy, particularly interventions that suppress this central neuroinflammation, represents a promising therapeutic strategy for restoring metabolic homeostasis and improving fertility in PCOS.

## Supplementary Information
