# Supplementary material for: Hypothalamic Inflammation as a Critical Link Between Metabolic Dysfunction and Impaired Endometrial Receptivity in Obese Polycystic Ovary Syndrome
Source: Physiol Res. 2026 Jun 1;75(3):485–97. doi: 10.33549/physiolres.935770 (PMC13557537; doi:10.33549/physiolres.935770)
Supplement: Supplementary file 1 [file 75_485_Suppl_Table_1.docx]

**Supplementary Table 1.** Western blot antibodies: dilution, supplier and catalogue number.

| *Antibodies* | **Species** | **Dilution** | **Supplier** | **Catalogue Number** |
| --- | --- | --- | --- | --- |
| *TLR4* | Rabbit | 1/500 | Affinity | AF7017 |
| *IKKβ* | Rabbit | 1/500 | Affinity | AF6009 |
| *NF-κB p65* | Rabbit | 1/1000 | CST | #8242 |
| *TNFα* | Rabbit | 1/500 | Affinity | AF7014 |
| *IL-10* | Rabbit | 1/500 | Affinity | DF6894 |
| *β-Actin* | Mouse | 1/2000 | Utibody | UM4001 |
| *Goat anti Rabbit IgG* | Goat | 1/4000 | Affinity | S0001 |
| *Goat anti Mouse IgG* | Goat | 1/4000 | Affinity | S0002 |

TLR4: toll-like receptor 4; IKKβ: IkB kinase β; NF-κB: nuclear factor kappa-B; TNFα: tumor necrosis factor α; IL-10: interleukin 10.
