# Supplementary material for: Hypothalamic Inflammation as a Critical Link Between Metabolic Dysfunction and Impaired Endometrial Receptivity in Obese Polycystic Ovary Syndrome
Source: Physiol Res. 2026 Jun 1;75(3):485–97. doi: 10.33549/physiolres.935770 (PMC13557537; doi:10.33549/physiolres.935770)
Supplement: Supplementary file 2 [file 75_485_Suppl_Table_2.docx]

**Supplementary Table 2.** Immunohistochemistry antibodies: dilution, supplier and catalogue number.

| *Samples* | **Antibodies** | **Species** | **Dilution** | **Supplier** | **Catalogue Number** |
| --- | --- | --- | --- | --- | --- |
| *Hypothalamus* | IBa-1 | Rabbit | 1/100 | FineTest | FNab04096 |
|  | HRP-conjugated Affinipure Goat Anti-Rabbit IgG (H+L) | Goat | 1/50 | FineTest | FNSA-0004 |
| *Uterus* | BMP2 | Rabbit | 1/400 | Bioss | bs-1012R |
|  | Goat Anti-Rabbit IgG Secondary Antibody (HRP) | Goat | 1/1500 | Sino Biological | SSA004 |

IBa-1: Ionic calcium binds adaptor molecule 1; BMP2: Bone morphogenetic protein 2.
