# Supplementary material for: Hypothalamic Inflammation as a Critical Link Between Metabolic Dysfunction and Impaired Endometrial Receptivity in Obese Polycystic Ovary Syndrome
Source: Physiol Res. 2026 Jun 1;75(3):485–97. doi: 10.33549/physiolres.935770 (PMC13557537; doi:10.33549/physiolres.935770)
Supplement: Supplementary file 3 [file 75_485_Suppl_Table_3.docx]

**Supplementary Table 3.** Immunofluorescence antibodies: dilution, supplier and catalogue number.

| *Antibodies* | **Species** | **Dilution** | **Supplier** | **Catalogue Number** |
| --- | --- | --- | --- | --- |
| *IBa-1* | Rabbit | 1/100 | FineTest | FNab04096 |
| *CD11b+CD1c* | Mouse | 1/200 | Abcam | Ab1211 |
| *HRP-conjugated Affinipure Goat Anti-Rabbit IgG (H+L)* | Goat | 1/50 | FineTest | FNSA-0004 |
| *Anti-mouse IgG (H+L), F(ab')2 Fragment (Alexa Fluor® 488 Conjugate)* | Goat | 1/1000 | CST | #4408 |
